# Identification and Functional Characterization of Glycosylation of Recombinant Human Platelet-Derived Growth Factor-BB in *Pichia pastoris*

**DOI:** 10.1371/journal.pone.0145419

**Published:** 2015-12-23

**Authors:** Mengmeng Dai, Changming Yu, Ting Fang, Ling Fu, Jing Wang, Jun Zhang, Jun Ren, Junjie Xu, Xiaopeng Zhang, Wei Chen

**Affiliations:** 1 Laboratory of Vaccine and Antibody Engineering, Beijing Institute of Biotechnology, Beijing, China; 2 Clinical Laboratory, The 148th Hospital of PLA, Zibo, China; Duke University Medical Center, UNITED STATES

## Abstract

Yeast *Pichia pastoris* is a widely used system for heterologous protein expression. However, post-translational modifications, especially glycosylation, usually impede pharmaceutical application of recombinant proteins because of unexpected alterations in protein structure and function. The aim of this study was to identify glycosylation sites on recombinant human platelet-derived growth factor-BB (rhPDGF-BB) secreted by *P*. *pastoris*, and investigate possible effects of O-linked glycans on PDGF-BB functional activity. PDGF-BB secreted by *P*. *pastoris* is very heterogeneous and contains multiple isoforms. We demonstrated that PDGF-BB was O-glycosylated during the secretion process and detected putative O-glycosylation sites using glycosylation staining and immunoblotting. By site-directed mutagenesis and high-resolution LC/MS analysis, we, for the first time, identified two threonine residues at the C-terminus as the major O-glycosylation sites on rhPDGF-BB produced in *P*. *pastoris*. Although O-glycosylation resulted in heterogeneous protein expression, the removal of glycosylation sites did not affect rhPDGF-BB mitogenic activity. In addition, the unglycosylated PDGF-BB^ΔGly^ mutant exhibited the immunogenicity comparable to that of the wild-type form. Furthermore, antiserum against PDGF-BB^ΔGly^ also recognized glycosylated PDGF-BB, indicating that protein immunogenicity was unaltered by glycosylation. These findings elucidate the effect of glycosylation on PDGF-BB structure and biological activity, and can potentially contribute to the design and production of homogeneously expressed unglycosylated or human-type glycosylated PDGF-BB in *P*. *pastoris* for pharmaceutical applications.

## Introduction

The yeast *Pichia pastoris* has been successfully used for the production of recombinant proteins because it combines the advantages of prokaryotic and mammalian cell expression systems [1]. The most important advantage over bacterial systems is that yeasts are able, like mammalian cells, to express eukaryotic proteins requiring post-translational modifications (PTMs) such as proteolytic cleavage, glycosylation, and disulfide bond formation [1–4].

Glycosylation is a very common PTM: it is estimated that 50–70% of human proteins are glycosylated [5]. Glycans contribute to protein folding, stability, immunogenicity, and biological functions and take part in many physiological processes [6–10]; thus, modification by glycosylation may be critical for the activity of therapeutic proteins [11–13]. There are two main types of glycosylation-related modifications in eukaryotic proteins: N-glycosylation, when oligosaccharides are covalently linked to asparagine at the consensus motif Asn-X-Thr/Ser (where X is any amino acid except Pro); and O-linked glycosylation, when oligosaccharides are attached to serine or threonine residues [10]. Similar to mammalian cells, yeasts can perform both N-linked and O-linked glycosylation, but the final glycosylation patterns are different [1, 2, 14, 15]. N-glycosylation in yeasts is characterized by hypermannosylation, and terminal α-1,3-mannose linkages result in short serum half-life or even immunogenicity of the recombinant glycoproteins, which significantly limits their therapeutic application in humans. Thus, efforts have been made to humanize yeast N-glycosylation, which has been accomplished in *P*. *pastoris* [2, 16].

In contrast to N-glycosylation, our understanding of O-glycosylation is limited. Unlike mammals with mucin-type O-glycosylation [17], yeasts use protein-O-mannosyltransferases (PMTs) to perform O-mannosylation [18, 19]. Protein O-mannosylation is conserved in prokaryotes and eukaryotes [15]; in mammals, it has been linked to the development of congenital muscular dystrophies [20–22]. In recent years, more concerns over the influence of O-glycosylation on the properties of recombinant therapeutic proteins have been raised. However, because of the deficit in effective specific O-glycosidases to hydrolyze the Man-α-O-Ser/Thr glycosidic bond [23], the identification of O-linked glycosylation and its effects on the activity of recombinant proteins expressed in *P*. *pastoris* require further investigation.

Platelet-derived growth factor subunit B (PDGF-B) is a member of a large and heterogeneous family of mitogenic factors characterized by a cysteine knot consisting of eight Cys residues. PDGF-B is produced by many cell types as a homodimer (PDGF-BB) formed through intra-molecular and inter-molecular disulfide bonds [24]; it is known to be involved in the process of wound healing [25], and is used as the active agent in the FDA-approved drug to treat diabetic foot ulcers [26].

We previously expressed secretable recombinant human PDGF-BB (rhPDGF-BB) in *P*. *pastoris* (unpublished work). However, rhPDGF-BB exhibits multiple isoforms, which complicates the identification and functional analysis of PTMs.

In this study, we demonstrated that O-glycosylation is the main PTM of the PDGF protein resulting in the heterogeneous expression pattern of PDGF-BB, and characterized O-glycosylation of rhPDGF-BB in detail. We found that rhPDGF-BB was modified by O-linked glycans at two major sites, which affected its affinity to a specific antibody. However, O-linked glycosylation did not interfere with PDGF-BB mitogenic activity and had no impact on its immunogenicity.

## Materials and Methods

### Cloning, expression, and purification of rhPDGF-BB

*PDGFB* genes encoding wild-type human PDGF-B (GenBank accession number NM_002608) or its mutant variants were synthesized by Sangon Biotech Co. Ltd (Shanghai, China) and cloned into the yeast expression vector pMEX9K using *Xho*I-*Eco*RI sites. The pMEX9k vector constructed in our laboratory has a backbone of the *P*. *pastoris* expression vector pPIC9k (Life Technologies, Carlsbad, CA, USA) lacking one *Xho*I site within the kanamycin resistance gene (the reference sequence of pPIC9k in GenBank: Z46234.1), so that the DNA fragment could be inserted into another *Xho*I site upstream of the Kex2 cleavage site and recombinant proteins with the native N-terminus could be produced. The resulting plasmids were linearized with *Sal*I and used to transform competent *P*. *pastoris* strain GS115 (Life Technologies) by electroporation, following the manufacturer’s instructions. For gene expression, recombinant yeast strains were grown in 5-L flasks containing 1 L Buffered Glycerol-complex medium (BMGY, prepared according to the user manual of the Multi-Copy Pichia Expression Kit; Invitrogen, Carlsbad, CA, USA) at 30°C, with constant shaking at 250 rpm, until the optical density at 600 nm reached 2. For expression induction, cells were harvested and resuspended in 1 L Buffered Methanol-complex medium (BMMY, prepared according to the user manual of the Multi-Copy Pichia Expression Kit) and grown at 28°C, with constant shaking at 220 rpm; 0.5% methanol (v/v) was added to the culture every 24 h during 72-h induction. Recombinant PDGF-BB and PDGF-BB^ΔGly^ were purified by hydrophobic interaction and ion exchange chromatography. Briefly, the supernatant was collected by centrifugation at 15,000 × *g* for 15 min, filtered through a 0.45-μm membrane, and loaded on an XK 26/20 column packed with Phenyl Sepharose 6 Fast Flow (High sub) (GE Healthcare, Fairfield, CT, USA) pre-equilibrated with 1 M (NH4)_2_SO_4_ in 20 mM phosphate buffer (PB), pH 7.2. The target protein was eluted with 50% ethanediol in 20 mM PB, pH 7.2, and applied to an XK26/20 column containing cation exchange Source 30S resin (GE Healthcare) equilibrated with 20 mM PB, pH 7.2. PDGF-BB was eluted with 1 M NaCl in PB, pH 7.2. Samples containing PDGF-BB were pooled and dialyzed against PBS (20 mM PB, 0.15 M NaCl, pH 7.2). After protein concentration was determined by the Lowry assay [27], purified samples were aliquoted and stored at -80°C.

### SDS-PAGE and western blotting

Purified proteins (5 μg) were mixed with sodium dodecyl sulfate polyacrylamide gel electrophoresis (SDS-PAGE) loading buffer with or without 20 mM dithiothreitol (DTT) and denatured at 100°C for 5 min. SDS-PAGE was performed using 4% stacking gels at 80 V for 15 min and 15% separating gels at 180 V for approximately 50 min (Mini-PROTEAN^®^ Tetra System, Bio-Rad, Hercules, CA, USA). After electrophoresis, the gels were stained with Coomassie Blue R250.

For western blotting, 0.1–0.5 μg proteins were separated as described above and transferred onto PVDF membranes (Immobilon-P, Millipore, Darmstadt, Germany) at 300 mA for 1 h (Mini-PROTEAN^®^ Tetra System; Bio-Rad). The membranes were then blocked with 5% non-fat milk in Tris-buffered saline with 0.05% Tween-20 (TBST, pH 7.6) for 1 h at room temperature, and PDGF-B was detected by incubation with a mouse anti-human PDGF-B antibody (1:1,000; sc-365805, Santa Cruz Biotechnology, Santa Cruz, CA, USA) for 1 h at room temperature. After washing with TBST three times, the membranes were incubated with an HRP-labeled anti-mouse secondary antibody (1:10,000; #7076, Cell Signaling Technology, Danvers, MA, USA) for 1 h at room temperature. Target proteins were visualized using a LAS4000 mini gel imaging system (GE Healthcare, Fairfield, CT, USA) after adding an HRP substrate (Immobilon; Millipore).

### N-terminal amino acid sequencing

Proteins separated by SDS-PAGE were transferred onto a PVDF membrane using 3-(cyclohexylamino)-1-propanesulfonic acid (CAPS) electroblotting buffer (239782-100GM, Merck, Darmstadt, Germany) for 1 h at 300 mA. The membrane was stained with 0.1% Coomassie Blue R250 to detect protein bands and then destained with 50% methanol. The areas containing protein bands were cut out, and the five amino acids at the N-termini of the proteins were sequenced by Edman degradation at the National Centre of Biomedical Analysis (Beijing, China).

### Protein glycosylation analysis

Proteins were treated with peptide N-glycosidase F (PNGase F, #P0704S, New England Biolabs, Ipswich, MA, USA), following the manufacturer’s protocol. Briefly, 9 μl of protein samples containing 10 μg or 20 μg purified protein was mixed with 1 μl of 10 × Glycoprotein Denaturing Buffer and denatured by heating at 100°C for 10 min. After cooling to room temperature, 3 μl NP-40, 3 μl G7 Reaction Buffer, 2 μl PNGase F, and 2 μl water were added, and the reaction mixture was incubated at 37°C for 3 h. After enzyme inactivation by heating at 100°C for 5 min, proteins were separated in a 15% (w/v) SDS-PAGE gel, followed by staining with Coomassie Blue R250 or the Glycoprotein Staining Kit (#24562, Thermo Fisher Scientific, Waltham, MA, USA), according to the manufacturer’s protocol. Recombinant human interferon-omega (rhIFN-ω) used as positive control for N-glycosidase F treatment has been previously expressed and purified in our laboratory.

### Liquid chromatography-mass spectrometry (LC/MS)

Recombinant PDGF and its mutant variants were reduced with DTT (2.5 mM) at 37°C for 30 min and diluted in buffer A (0.1% formic acid in water) to 10 ng/μl prior to LC/MS analysis. Proteins were separated on the Easy-spray column (15 cm × 75 μm ID, 3-μm C18 particles) using the EASY-nLC system (Thermo Fisher Scientific). A linear gradient of buffer B (0.1% formic acid in methanol; 0–90% in 20 min) was applied at the flow rate of 300 nl/min. High resolution spectra were acquired with a resolution of 60,000 at m/z 350–1600 using a Q Exactive Mass Spectrometer (Thermo Fisher Scientific) and were deconvoluted using the Xtract software (Thermo Scientific).

### Biological activity assay

BALB/C 3T3 cells (Beijing Cell Resource Centre, Beijing, China) were cultured in Dulbecco's Modified Eagle’s medium (DMEM; Life Technologies) containing 10% (v/v) fetal bovine serum (FBS; Life Technologies) at 37°C in a humidified 5%-CO_2_ incubator. For cell proliferation assay, BALB/C 3T3 cells were seeded in a 96-well plate at a density of 5 × 10^3^ cells per well in 100 μl of 10% FBS-containing DMEM for 24 h. The medium was replaced with 0.4% FBS-containing DMEM for another 24 h, and then 100 μl fresh 0.4% FBS- containing DMEM with two-fold serial dilutions of 100 ng/ml PDGF-BB were added for 64–72 h, and cell proliferation was determined by the water-soluble tetrazolium (WST)-1 assay; each dilution was analyzed in triplicate. Briefly, 10 μl of WST-1 solution (11644807001, Roche, Basel, Switzerland) was added into each well, and after 3 h of incubation, the absorbance at the test wavelength of 450 nm and reference wavelength of 630 nm was measured using the SpectraMax^®^ Paradigm^®^ Multi-Mode Detection Platform (Molecular Devices, Sunnyvale, CA, USA). The data were analyzed by the nonlinear regression sigmoidal dose-response analysis with variable slopes using the GraphPad Prism 6 software. The assay was carried out in triplicate and repeated three times independently.

### Immunogenicity analysis

Animal studies were approved by the Institutional Animal Care Committee of Beijing Institute of Biotechnology (IACUC permit number: AMMS-08-2014-002). Female BALB/C mice (6–8 weeks old) were purchased from Laboratory Animal Centre of the National Institute for the Control of Pharmaceutical and Biological Products (Beijing, China) and were divided into three groups: PDGF-BB group (n = 8), PDGF-BB^ΔGly^ group (n = 8), and PBS control group (n = 6). Mice were injected with 50 μg PDGF-BB or PDGF-BB^ΔGly^ in 100 μl PBS, or with 100 μl PBS, respectively, via the tail vein. Boost immunizations were performed with the same protein dose 2 weeks later. Mice were bled 1, 3, and 4 weeks after the first immunization, and the titers of antibodies against PDGF-BB or PDGF-BB^ΔGly^ were determined by ELISA. Briefly, the antigens were diluted to 2 μg/ml with coating buffer (0.05 M carbonate buffer, pH 9.6), and 100 μl per well of the solution was added to 96-well plates and incubated overnight at 4°C. Following three washes with PBS containing Tween-20 (PBST), 150 μl of 3% BSA in PBST was added to each well to block non-specific binding for 1 h at 37°C; then, two-fold serial dilutions of each serum sample were added for 1 h at 37°C. After three washes with PBST, an HRP-conjugated anti-mouse secondary antibody (1:10,000, #7076; Cell Signaling Technology) was added in 100 μl per well for 1 h at 37°C. The reaction was developed with 100 μl per well of TMB substrate (PR1200, Solarbio, China) at room temperature for 10 min and stopped by adding 50 μl 2 M sulfuric acid solution; the absorbance was measured with a test wavelength of 450 nm and a reference wavelength of 630 nm. The antibody titer was considered positive if the OD value was not less than the mean OD of the negative samples (PBS-immunized mice) plus three standard deviations.

### Statistical analysis

The data were analyzed with the unpaired two-tailed t-test using the GraphPad Prism6 software. Two-way ANOVA followed by multiple comparisons with Fisher’s LSD method was used for antibody cross-reactivity analysis. P < 0.05 was considered statistically significant.

## Results

### rhPDGF-BB secreted by *P*. *pastoris* was modified by O-glycosylation

**Fig 1:**
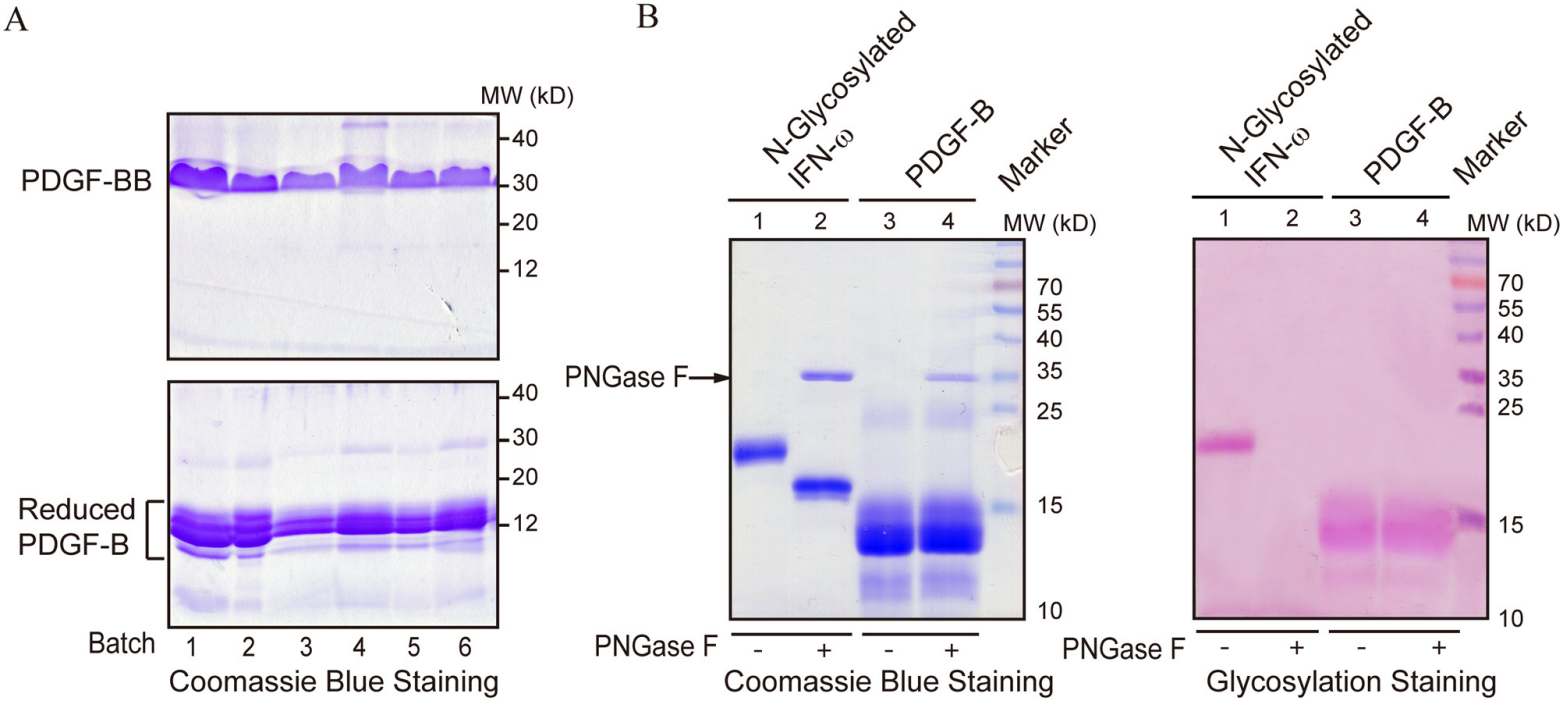
Heterogeneous expression of glycosylated recombinant human PDGF-BB in *Pichia pastoris*. (A) Purified rhPDGF-BB proteins from six independent expression and purification experiments were analyzed by SDS-PAGE. Heterogeneous rhPDGF-BB bands could be observed under reducing conditions. (B) Purified rhPDGF-BB and rhIFN-ω produced by *P*. *pastoris* were treated with PNGase F to hydrolyze N-glycan residues. Two gels loaded with the same amount of each protein (5 μg) were simultaneously subjected to SDS-PAGE, and analyzed by Coomassie Blue staining (left panel) and glycosylation staining (right panel), respectively. There were no differences between rhPDGF-BB samples treated or not with PNGase F.

Mature PDGF-B consists of 109 amino acids (Ser82–Thr190; NCBI Reference Sequence: NP_002599.1). In this study, we expressed the PDGF-B fragment Thr87–Thr190, because five N-terminal amino acids are usually cleaved in *P*. *pastoris* (data not shown). The 104-amino acid fragment was cloned into the pMEX9k vector, where rhPDGF-B expression was controlled by the alcohol oxidase 1 (AOX1) promoter inducible by methanol. Under reducing conditions, purified rhPDGF-B appeared in an SDS-PAGE gel as a group of bands with molecular weights between 10 and 15 kDa (Fig 1A, lower panel), whereas under non-reducing conditions, PDGF-BB demonstrated a single protein band with a molecular weight of about 30 kDa (Fig 1A, upper panel). The glycosylation status of rhPDGF-BB was examined in the reaction with PNGase F, which hydrolyzes *N*-linked glycans [28]; rhIFN-ω, an N-glycosylated protein expressed in *P*. *pastoris*, was used as a control. Coomassie Blue staining of the hydrolyzed products resolved by reducing SDS-PAGE showed that PNGase F treatment decreased the molecular weight of IFN-ω, presumably because of the removal of N-glycans (Fig 1B, left panel, lanes 1–2), but did not affect that of PDGF-B (Fig 1B, left panel, lanes 3–4). Consistent with this results, glycoprotein staining patterns of PDGF-B treated or not with PNGase F were similar (Fig 1B, right panel, lanes 3–4), whereas PNGase F-treated IFN-ω could not be detected by glycoprotein staining (Fig 1B, right panel, lanes 1–2). Because PNGase F is specific for N-linked glycosylation, these results indicate that secreted rhPDGF-BB did not contain N-glycans, which is consistent with the absence of potential N-glycosylation sites in mature PDGF-B, as evidenced by the analysis using the CBS network tool (NetNGlyc 1.0 Server; http://www.cbs.dtu.dk/services/NetNGlyc/) (S1 Fig).

### rhPDGF-BB heterogeneity was a result of O-linked glycosylation and proteolysis at Arg32-Thr33

**Fig 2:**
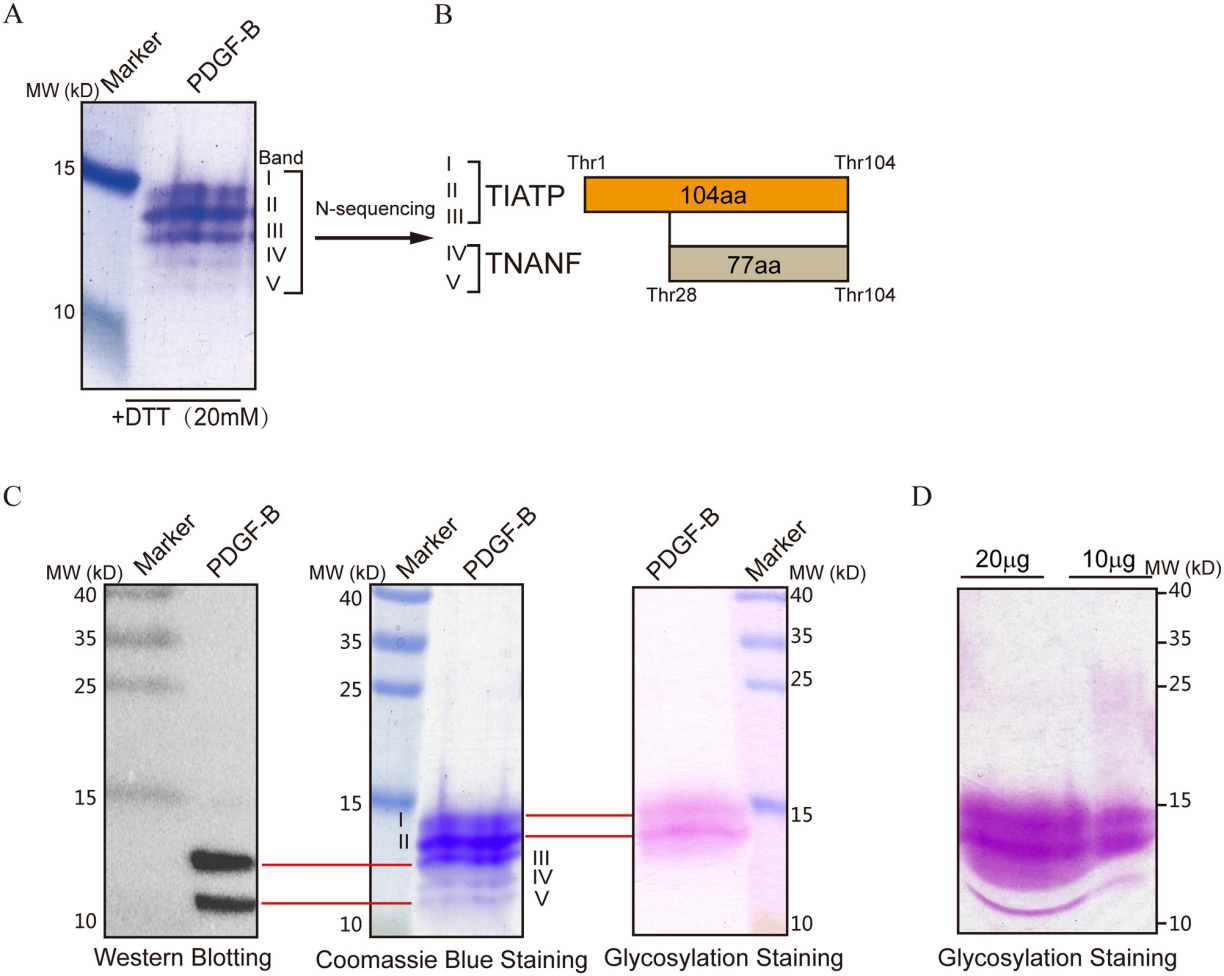
Identification of rhPDGF-BB isoforms. (A) Five isoforms detected by SDS-PAGE were transferred to a PVDF membrane for protein N-terminal sequencing. (B) Schematic representation of N-terminal sequencing results. Bands I, II, and III represent intact PDGF-BB, while bands IV and V are truncated isoforms generated by the cleavage at the Arg 27-Thr 28 site. (C) rhPDGF-BB (5 μg) was subjected to SDS-PAGE under reducing conditions and analyzed by western blotting, Coomassie Blue staining, and glycosylation staining, respectively. Two bands corresponding to isoforms III and IV stained by Coomassie Blue could be detected by antibody staining (marked with red lines). Glycosylation staining revealed another two bands corresponding to isoforms I and II (marked with red lines). (D) SDS-PAGE and subsequent glycosylation staining of higher rhPDGF-BB load (10 μg and 20 μg) revealed three bands; the third band with the lowest molecular weight was assumed to correspond to isoform IV.

In the absence of N-glycosylation, heterogeneous pattern of rhPDGF-B expression indicated by multiple bands in the SDS-PAGE gel (Fig 1) suggested possible O-glycosylation. We then attempted to identify each of the five bands detected by Coomassie Blue staining of reducing SDS-PAGE gels (Fig 2A) by determining their N-terminal amino acid sequences using Edman degradation analysis. The results showed that the first five amino acid residues of bands I, II, and III were Thr-Ile-Ala-Glu-Pro, corresponding to those of the native PDGF-B, whereas those of bands IV and V were Thr-Asn-Ala-Asn-Phe, indicating that these bands represented two distinct truncated fragments were generated by the cleavage between Arg 27 and Thr 28 (Fig 2B).

We then performed western blot analysis using a monoclonal antibody (F-3) against the C-terminal sequence of PDGF-B, which detected only two PDGF isoforms (Fig 2C, left panel) corresponding to bands III and V in the Coomassie Blue-stained gel (Fig 2C, middle panel). The absence of the antibody reaction with isoforms I, II, and IV could not be due to C-terminal degradation, because their molecular weights were higher than those of isoforms III or V were. A more plausible explanation is that the antibody recognition epitope was hindered by modification and thus inaccessible for binding. This notion was supported by glycoprotein staining analysis, which detected protein fragments corresponding to bands I, II, and IV but not those corresponding to bands III and V (Fig 2C, right panel, and Fig 2D). These data suggest that O-glycosylation at the C-terminus changed the antigenicity of PDGF-BB and that the heterogeneous expression pattern of PDGF-BB was due to glycosylation and proteolysis. The identities of PDGF-BB isoforms are presented in Table 1.

**Table 1:** Deductive identifications of PDGF-BB isoforms.

Isoform	Identification
**I**	Intact PDGF-BB with O-linked glycosylation
**II**	Intact PDGF-BB with O-linked glycosylation
**III**	Intact PDGF-BB
**IV**	Truncated PDGF-BB with O-linked glycosylation
**V**	Truncated PDGF-BB

### Analysis of O-glycosylation sites

**Fig 3:**
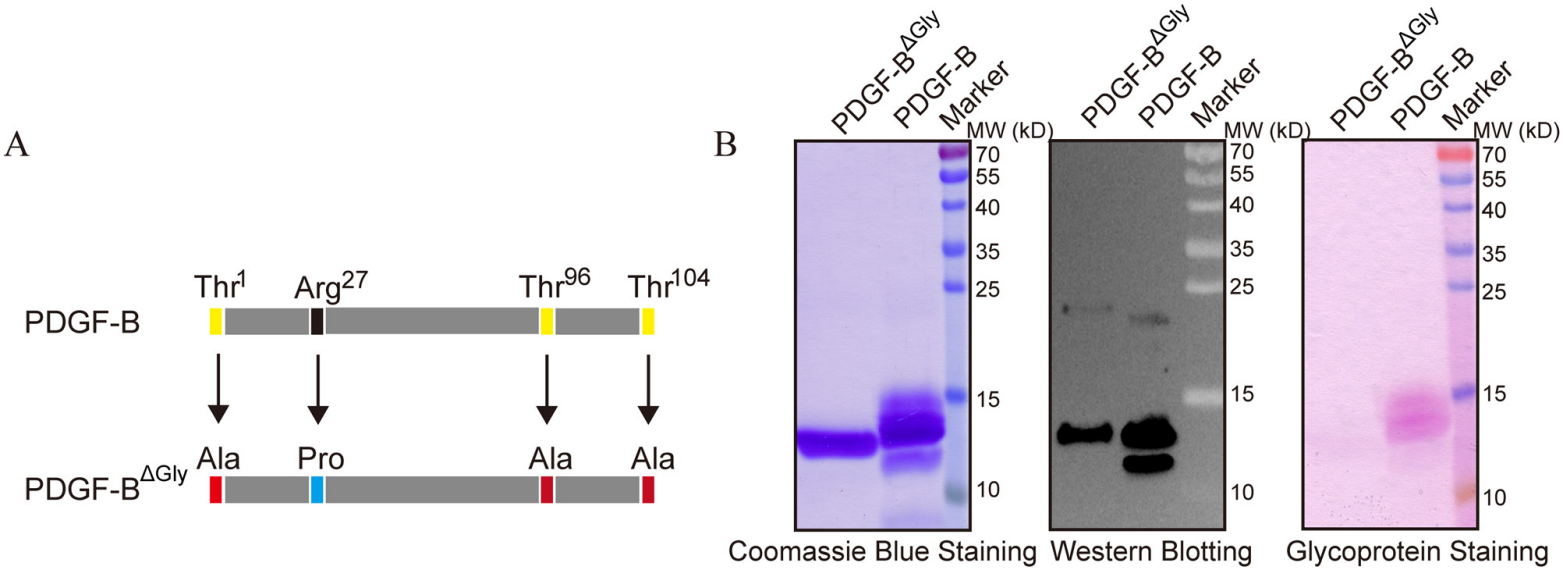
Mutation of the putative O-glycosylation sites in rhPDGF-BB. (A) Schematic representation of point mutations in PDGF-BB. (B) Purified wild-type PDGF-BB and PDGF-BB^ΔGly^ mutant were separated by reducing SDS-PAGE and analyzed by Coomassie Blue staining (left panel), western blotting (middle panel), and glycosylation staining (right panel). A single homogeneous band could be observed after western blotting and Coomassie Blue staining, while no band was detected by glycosylation staining of the mutant.

To perform functional analysis, we needed to determine O-glycosylation sites on PDGF-B. These sites are difficult to predict because in contrast to N-glycosylation, there are no O-glycosylation consensus motifs, and any Thr or Ser can be a potential target [10]. PDGF-B sequence of 104 amino acids contains nine Thr and two Ser residues, which could be O-glycosylated. However, we speculated that at least one O-glycosylation site should be at the C-terminus, as evidenced by glycosylation staining of isoforms I, II, and IV (Fig 2C). In addition, the N-terminal amino acids 1–27 was also expected to contain one O-glycosylation site, because the truncated PDGF lacking the N-terminus had only one glycosylated isoform (IV), but the full-length protein had two (I, II). Based on this reasoning and the bioinformatics analysis (NetOGlyc 3.1 Server; http://www.cbs.dtu.dk/services/NetOGlyc-3.1/) (S2 Fig), we predicted three Thr residues as potential O-glycosylation sites (Fig 3A).

To test the predicted sites, we performed site-directed mutagenesis to substitute the three putative Thr residues (Thr 1, Thr 96, and Thr 104) with Ala; in addition, Arg27 was replaced by Pro to remove the proteolysis site, which does not affect the activity of PDGF-BB [29]. The mutated PDGF-B (PDGF-BB^ΔGly^, M1) was expressed in *P*. *pastoris*, as described for PDGF-B, and the purified protein was analyzed by SDS-PAGE. Under reducing conditions, only one homogeneous band was detected by both Coomassie Blue staining (Fig 3B, left panel) and western blotting (Fig 3B, middle panel), while glycosylation staining was negative (Fig 3B, right panel), indicating that mutagenesis removed O-glycosylation sites.

**Fig 4:**
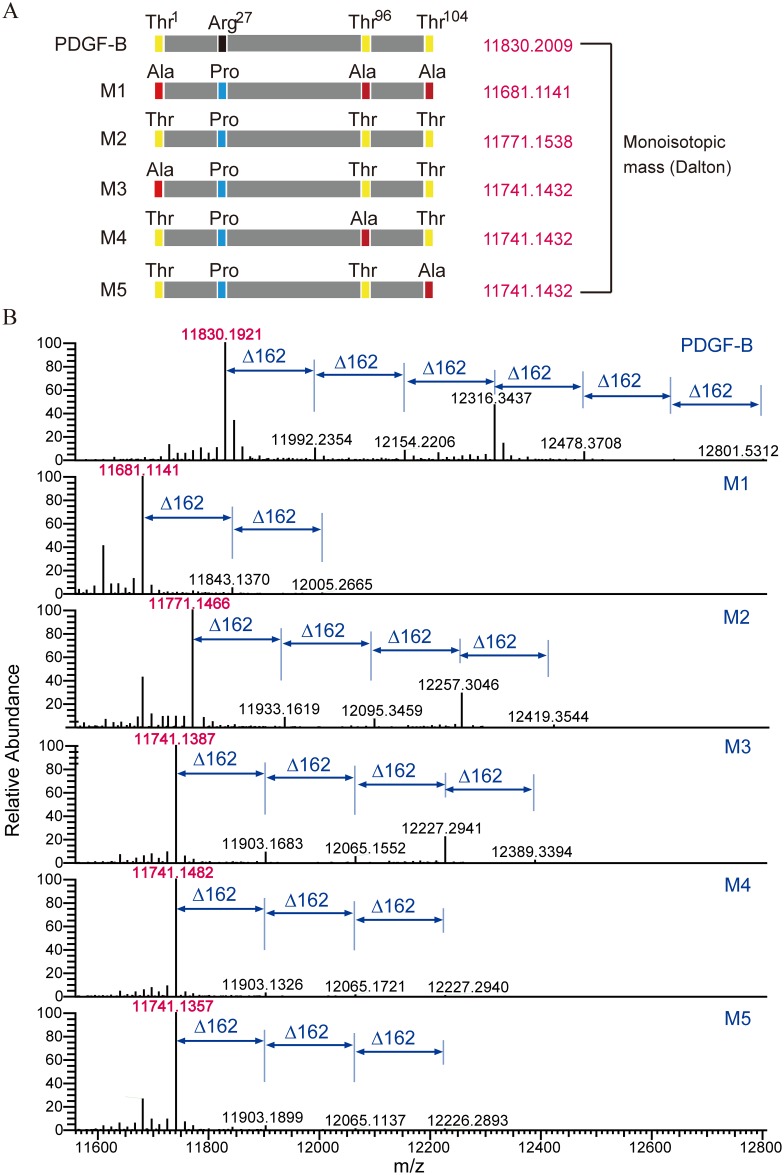
Identification of O-glycosylation sites in rhPDGF-BB using LC/MS. (A) Schematic representation of PDGF-B mutants. Monoisotopic mass of every mutant is shown. (B) Deconvoluted mass spectra of the wild-type PDGF and its mutants are presented with monoisotopic peaks; glycosylated isoforms are annotated.

To further characterize PDGF-B O-glycosylation sites, we constructed four additional PDGF-B mutants: M2 carried Arg27Pro mutation, and M3–M5 harbored Arg27Pro and one of three Thr-to-Ala substitutions (Fig 4A). The analysis of PDGF-B wild-type and M1–M2 mutants by high-resolution LC/MS showed that different isoforms with up to six sugar residues could be detected in the wild-type PDGF; isoforms with three sugar residues were the most abundant (Fig 4B, PDGF-B). In contrast, glycosylation was scarcely detected in PDGF-BB^ΔGly^ (Fig 4B, M1), which is in accordance with the results presented in Fig 3B. The spectrum patterns of M2 and M3 were similar, demonstrating that Thr1 is not a major PDGF site targeted by O-glycosylation in *P*. *pastoris*. However, the mutation of Thr96 or Thr104 eliminated most of glycosylated PDGF isoforms, especially those with three sugar residues (Fig 4B, M4 and M5). These data confirmed that Thr96 and Thr104 in PDGF-B are the major sites targeted by O-glycosylation in *P*. *pastoris*, while O-glycosylation at Thr1 is rare (Fig 4B).

### O-linked glycosylation did not affect PDGF-BB biological activity

**Fig 5:**
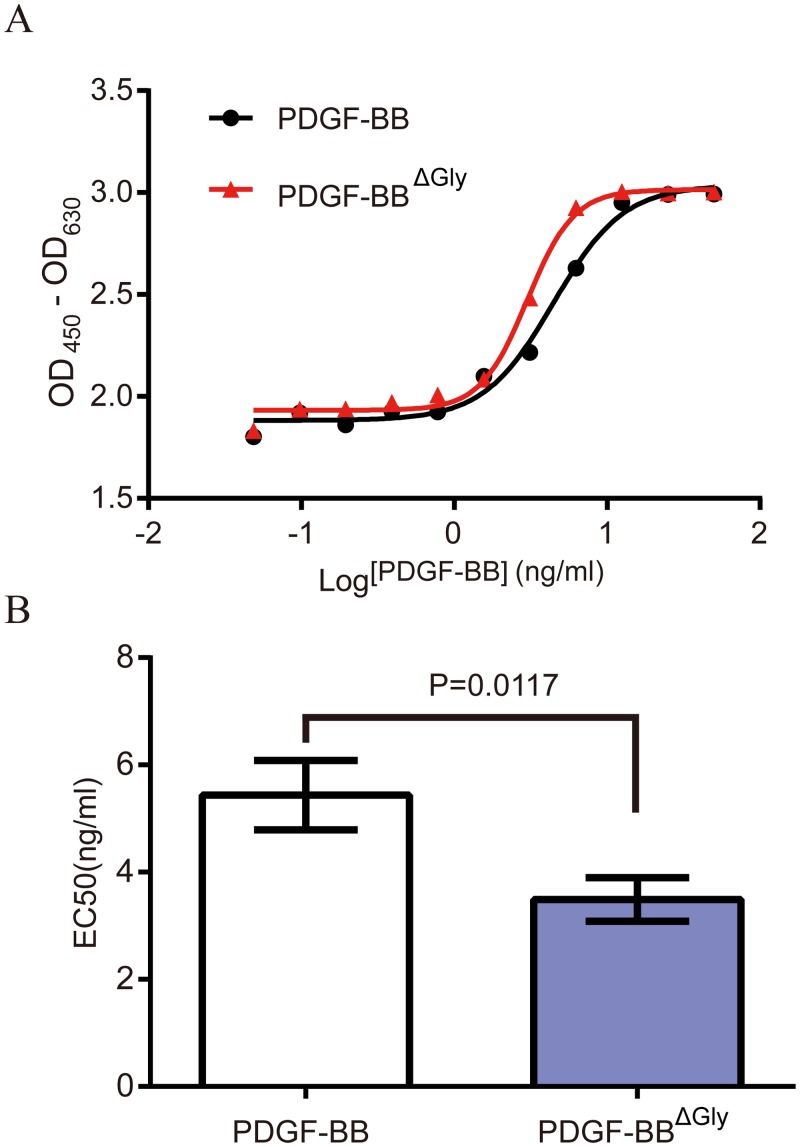
Mitogenic activity of rhPDGF-BB and PDGF-BB^ΔGly^. (A) Proliferation of BALB/C 3T3 cells stimulated with different concentrations of rhPDGF-BB (black line) or PDGF-BB^ΔGly^ (red line) was analyzed by the WST-1 assay. Dose-response curves of a representative assay are shown. (B) EC_50_ values were calculated based on three independent experiments and expressed as the mean ± SD (P = 0.0117).

The observation that O-glycosylation at PDGF-B C-terminus hindered the antibody-specific epitope suggested that this modification could affect PDGF-BB functional activity. To address this possibility, we tested the mitogenic activity of PDGF-BB and PDGF-BB^ΔGly^ (M1) by stimulating BALB/C 3T3 cells and measuring their proliferation by the WST-1 assay, a version of the classical MTT assay [30]. The results indicated that both PDGF-BB and PDGF-BB^ΔGly^ were able to stimulate the proliferation of BALB/C 3T3 cells (Fig 5A).

Four Parameter Logistic fitness and the 50% effective concentrations (EC_50_) were calculated using the GraphPad software. The results of three independent assays showed that the EC_50_ values for PDGF-BB and PDGF-BB^ΔGly^ were 5.434 ± 0.6475 ng/ml and 3.492 ± 0.4078 ng/ml, respectively (Fig 5B). Differences in mitogenic activity between PDGF- BB and PDGF-BB^ΔGly^ may be explained by the presence of truncated isoforms in the PDGF-BB samples, which lost the key residues for receptor activation [29].

### O-glycosylation did not influence PDGF-BB immunogenicity

**Fig 6:**
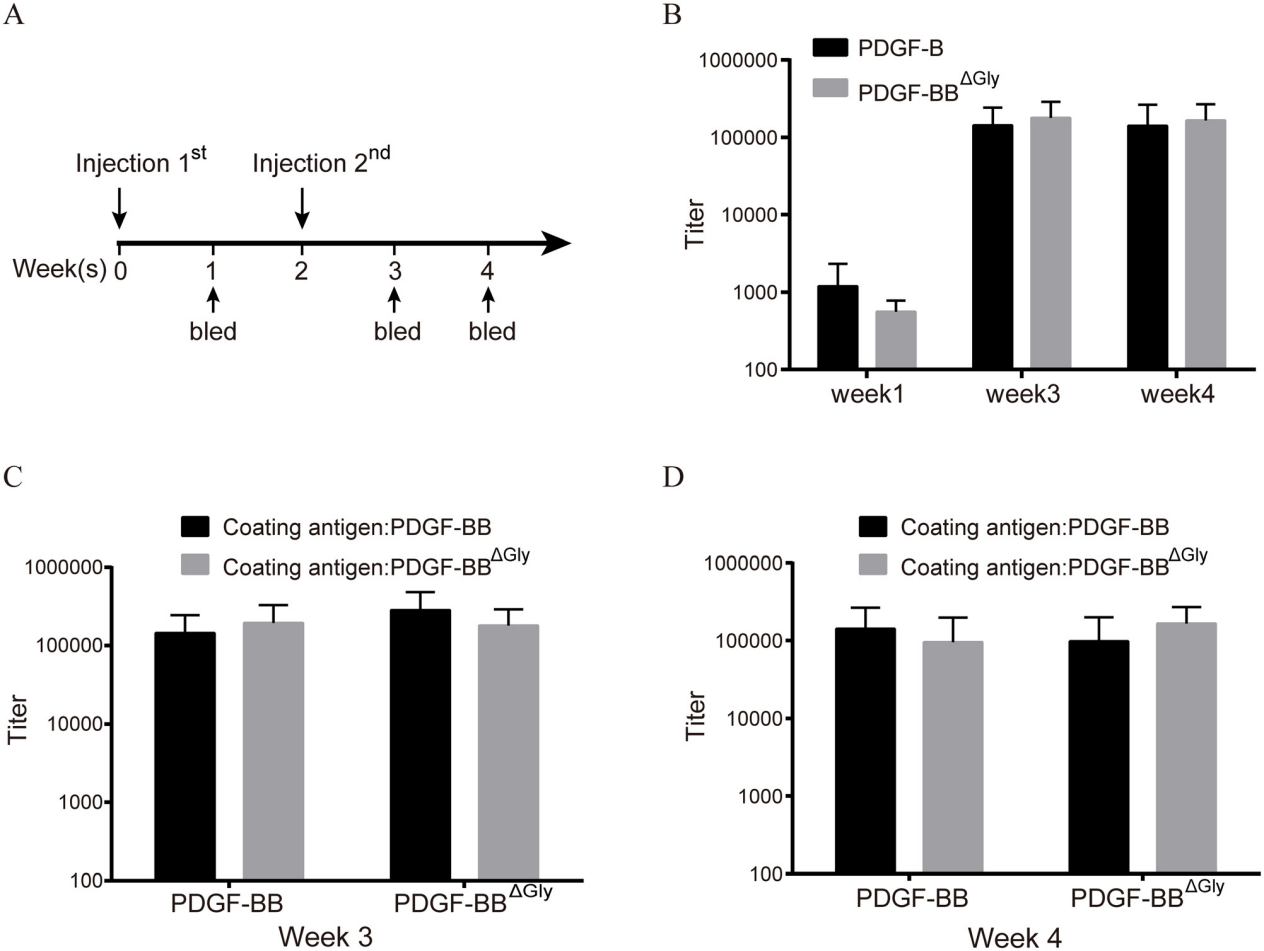
Mouse immunization with rhPDGF-BB and PDGF-BB^ΔGly^. PDGF-BB- and PDGF-BB^ΔGly^-specific antibody titers presented as the mean ± SD are defined as the reciprocal endpoint dilution. (A) Schematic representation of the immunization procedure. (B) Serum antibody titers against PDGF-BB and PDGF-BB^ΔGly^ in mice boosted at week 1, week 3, and week 4 after the first immunization (week 1, P = 0.25; week 3, P = 0.51; week 4, P = 0.68). (C, D) The titers of antisera against PDGF-BB and PDGF-BB^ΔGly^ at week 3 (C) and week 4 (D).

The immunogenicity of recombinant proteins expressed in *P*. *pastoris* can be modulated by high-mannose glycans, which can bind to dendritic cell-specific Intercellular Adhesion Molecule 3-grabbing Nonintegrin (DC-SIGN) [31–33]. It has also been reported that N-linked and O-linked glycans generated in *P*. *pastoris* have different effects on the immunogenicity of recombinant vaccines [9]. To address this issue, we immunized mice twice via the tail vein with 50 μg PDGF-BB or PDGF-BB^ΔGly^ (Fig 6A) and measured serum antibody titers at different post-injection time points by ELISA. It was observed that antibody response was boosted rapidly after the second immunization with both PDGF-BB and PDGF-BB^ΔGly^, and the titers were similar (P > 0.05 for every time point; Fig 6B). In addition, to assess possible generation of O-glycan-specific antibodies, we tested antiserum cross-reactivity. The results indicated that PDGF-BB antiserum recognized PDGF-BB^ΔGly^ (week 3, P = 0.48; week 4, P = 0.39) and vice versa (week 3, P = 0.16; week 4, P = 0.22) (Fig 6C and 6D). These data suggest that O-linked glycosylation occurring in *P*. *pastoris* did not change the immunogenicity of the rhPDGF-BB protein.

## Discussion

In this study, we identified two major O-glycosylated sites of rhPDGF-BB expressed in *P*. *pastoris*. We showed that O-glycosylation resulted in heterogeneous expression pattern of PDGF-BB, but did not interfere with its functional activity or immunogenicity.

The challenge in the identification of O-glycosylated residues was posed by the complicated PDGF-B expression pattern (at least five isoforms were detected by SDS-PAGE) and multiple potential glycosylation sites (nine Thr and two Ser residues). We analyzed glycosylation spectra with electron-transfer dissociation (ETD) coupled with MS/MS, which suggested that at least six Thr residues could be O-glycosylated (data not shown). However, even with this information, it was not possible to evaluate the contribution of each Thr residue to overall PDGF-B glycosylation. In this study, we assessed the structural and functional roles of three Thr sites by performing step-wise site-directed mutagenesis and comparing the mutants with the wild-type PDGF-B in terms of glycosylation patterns, immunogenicity, and mitogenic activity.

One interesting finding is that the mutation of either Thr94 or Thr104 abolished O-glycosylation on the other site. The underlying mechanism is unclear, but likely involved conformational changes induced by residue substitution. Together with the observation that glycosylation can obstruct access to PDGF antibody-binding site, these data suggest that the C-terminal Thr94–Thr104 region in PDGF-B plays an important role in defining the structure and function of this protein. Interestingly, this nine-amino acid fragment presents structural distinction between PDGF and transforming growth factor β (TGF-β) which both belong to the cystine-knot superfamily but have different functions [34].

Although O-glycosylation is involved in the immune response, we did not detect any significant difference in immunogenicity between PDGF-BB and its glycosylation-deficient mutant PDGF-BB^ΔGly^, although the most abundant triple-glycosylated isoform constituted almost 30% of total PDGF molecules, as evidenced by the MS data (Fig 4B, PDGF-B). One possible reason is that *P*. *pastoris* is deficient in highly immunogenic terminal α-1,3-mannosyl groups, because the gene encoding α-1,3-mannosyltransferase was not detected by genome sequencing [19].

Another reason for which no obvious effects of O-glycosylation were observed in this study may be the short length of O-oligosaccharide chains synthesized in *P*. *pastoris*. We did not detect more than six sugar monomers, which is consistent with the fact that in *P*. *pastoris*, the major secreted proteins—which are O-mannosylated—have a maximum of six mannose residues, while in mammals, O-mannosyl glycans are more complex [15]. Thus, *P*. *pastoris*-expressed proteins carrying short-chain O-oligosaccharides may be unable to effectively induce specific immune responses, as evidenced by our immunization experiments.

Glycoengineering of *P*. *pastoris* to target O-glycosylation for biopharmaceutical applications has been a focus of recent research [18, 35]. The removal of O-linked mannose from *P*. *pastoris*-expressed proteins by enzymatic treatment has also been investigated [23, 36]. However, these efforts should be based on clear understanding of the effects of O-glycosylation on recombinant proteins. While a decrease in biological activity or increase in immunogenicity is undesirable for therapeutic peptides such as PDGF-BB, the enhancement of immunogenicity via O-glycosylation would be beneficial for proteins used as vaccine components [32]. However, the possibility that O-glycans may hinder recognition epitopes and reduce the production of neutralizing antibodies should also be considered. This is supported by the present findings that O-glycosylation affected the interaction of PDGF-BB and its C-terminus-specific antibody.

In summary, we determined that PDGF-B produced by *P*. *pastoris* is O-glycosylated. Although the structure of mannan oligosaccharides should be further analyzed, this work has provided direct evidence that *P*. *pastoris* can produce more homogeneous, deglycosylated PDGF-BB with the activity and immunogenicity comparable to those of the wild type protein. Although O-glycosylation does not affect the biological activity or immunogenicity of PDGF-BB, it leads to the heterogeneous expression pattern, which would hamper biopharmaceutical processing and reproducibility [37, 38]. Therefore, the PDGF-BB glycosylation mutant with enhanced homogeneity described in this study may have potential practical applications in the pharmaceutical industry.

## Supporting Information

S1 Fig Prediction of N-glycosylation sites in PDGF-B using the NetNGlyc 1.0 Server. No N-glycosylation sites (Asn-X-Thr/Ser) were found (default threshold = 0.5).
(TIF)

S2 Fig Prediction of O-glycosylation sites in PDGF-B using the NetOGlyc 3.1 Server. Three Thr residues, Thr 1, Thr 96, and Thr 104, were predicted as potential O-glycosylation sites (default threshold = 0.5).
(TIF)
