# Supplementary figures and images for: Identification and Functional Characterization of Glycosylation of Recombinant Human Platelet-Derived Growth Factor-BB in Pichia pastoris
Source: PLoS One. 2015 Dec 23;10(12):e0145419. doi: 10.1371/journal.pone.0145419 (PMC4689512; doi:10.1371/journal.pone.0145419)

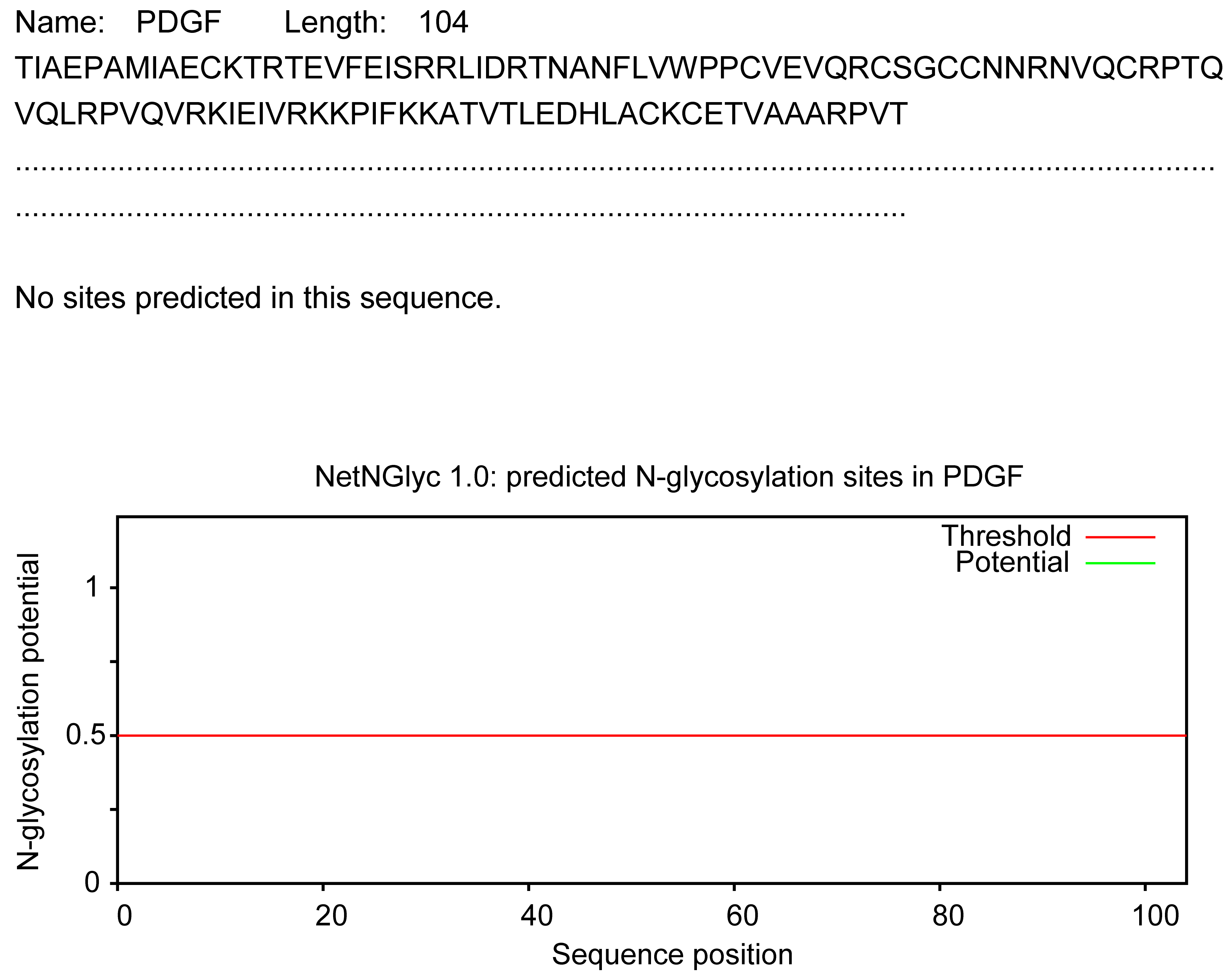

Supplement: S1 Fig — No N-glycosylation sites (Asn-X-Thr/Ser) were found (default threshold = 0.5). (TIF) [file pone.0145419.s001.tif]

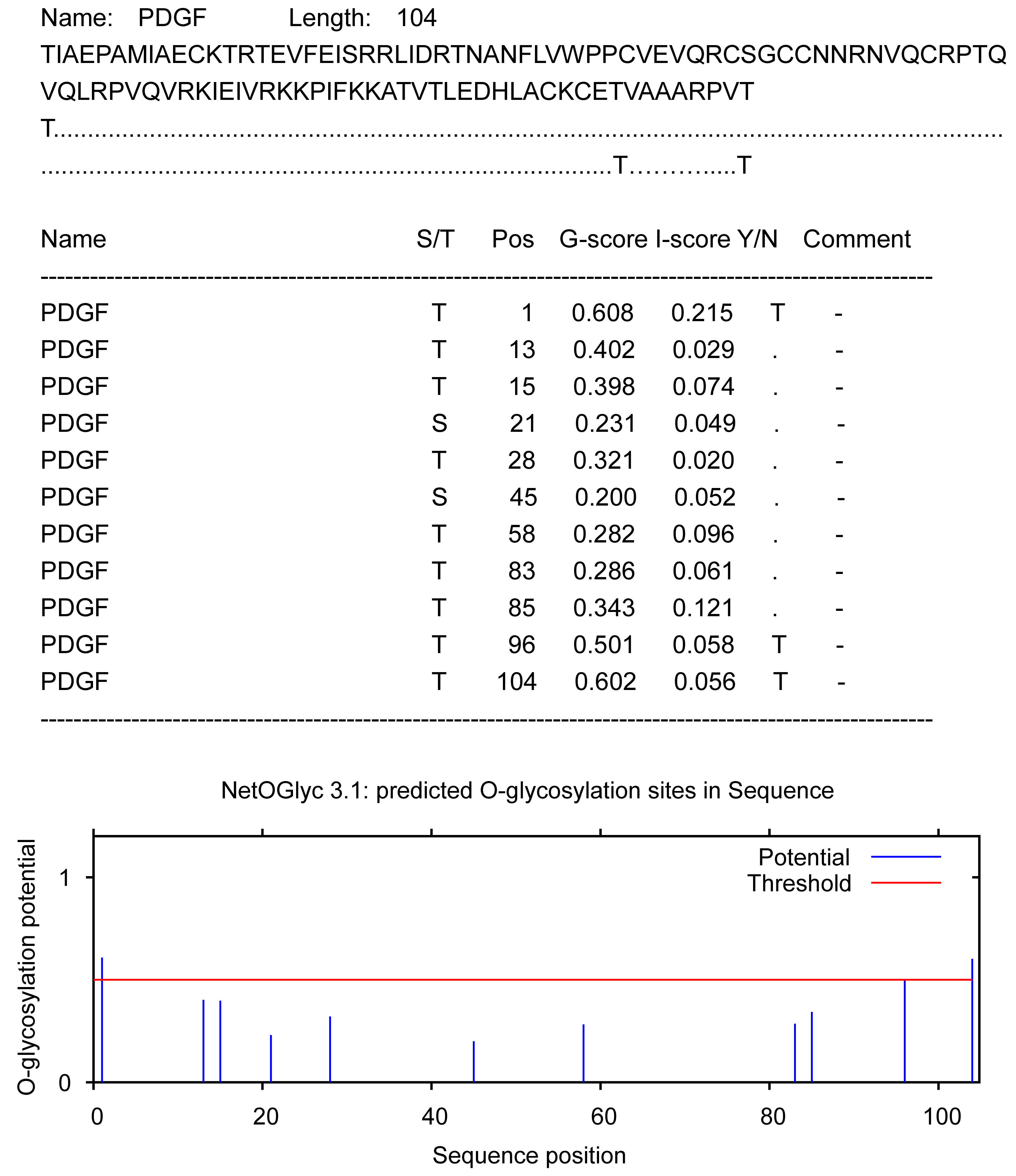

Supplement: S2 Fig — Three Thr residues, Thr 1, Thr 96, and Thr 104, were predicted as potential O-glycosylation sites (default threshold = 0.5). (TIF) [file pone.0145419.s002.tif]
